# Incidence and outcomes of subsequent malignancy after allogeneic hematopoietic stem cell transplantation in adult patients with severe aplastic anemia

**DOI:** 10.1007/s44313-024-00046-2

**Published:** 2024-12-24

**Authors:** Daehun Kwag, Sung-Soo Park, Sung-Eun Lee, Hee-Je Kim, Jong Wook Lee

**Affiliations:** 1 Department of Hematology, College of Medicine, Seoul St. Mary’s Hospital, The Catholic University of Korea, Seoul, Republic of Korea; 2 Division of Hematology-Oncology, Hanyang University Seoul Hospital, Seoul, Republic of Korea

**Keywords:** Aplastic anemia, Subsequent malignancy, Allogeneic hematopoietic transplantation

## Abstract

**Purpose:**

This study investigated the occurrence of subsequent malignancies (SM) in adult patients with severe aplastic anemia (SAA) after allogeneic hematopoietic stem cell transplantation (allo-HSCT) to address the lack of large-scale, long-term data on this complication.

**Methods:**

A retrospective cohort analysis of 376 adult patients with SAA who underwent allo-HSCT between 2002 and 2021 at a single center was conducted. The incidence, risk factors, and survival impact of SM were also examined.

**Results:**

During the follow-up period, 31 cases of SM (8.2%) were identified. Approximately one-third (32.3%) of SM cases were hematologic malignancies, including post-transplant lymphoproliferative disorder (16.1%), myelodysplastic neoplasm (6.5%), and acute myeloid leukemia (3.2%). Solid tumors accounted for 67.7% of cases, with thyroid cancer being the most prevalent (25.8%). The 15-year cumulative incidence of SM was 11.2%, and the hazard ratio for overall survival according to the development of SM was 16.25 (*p* < 0.001). High-dose total body irradiation (TBI), anti-thymocyte globulin (ATG), and moderate-to-severe chronic graft-versus-host disease (GVHD) were identified as significant risk factors for subsequent malignancy. Post-transplant SAA patients exhibited a 3.54-fold higher observed cancer incidence than the expected incidence calculated from the age-, sex-, and calendar year-matched general population.

**Conclusion:**

SM is a significant long-term complication in patients with posttransplant SAA and has a substantial survival impact. Patients receiving high-dose TBI or ATG, and those with moderate-to-severe chronic GVHD, require vigilant long-term monitoring.

**Supplementary Information:**

The online version contains supplementary material available at 10.1007/s44313-024-00046-2.

## Introduction

Aplastic anemia is a life-threatening condition characterized by bone marrow destruction that leads to a deficiency in the production of red blood cells, white blood cells, and platelets [1]. Allogeneic hematopoietic stem cell transplantation (allo-HSCT) remains the most effective treatment for severe aplastic anemia (SAA), especially in patients who do not respond to immunosuppressive therapy (IST) [1, 2]. Over the past few decades, the posttransplant survival of patients with SAA has steadily improved, with long-term post-HSCT survival rates exceeding 80% since the 2000s [3]. However, as survival time after HSCT increases, patients are increasingly at risk of developing late complications, including the development of subsequent malignancies (SMs) [4]. Previous studies have reported that the incidence of SMs is 2–10 times higher in HSCT recipients than in age-matched controls [5–7].

Although several studies have attempted to determine the incidence of SM, risk factors for SM development, and the impact of SM on post-transplant outcomes, most of these studies have focused on patients with hematological malignancies. Indeed, given the paucity of data on SAA incidence, even in multicenter or nationwide studies tracking the development of SM in allo-HSCT recipients, the proportion of patients with bone marrow failure, including SAA, is low [8–11]. The non-malignant nature and long-term survival of SAA make the investigation of SM more important than other HSCT complications [12]. Paradoxically, there is a lack of research in this area. Therefore, this study aimed to investigate the unmet needs by identifying the incidence, type, risk factors, and survival outcomes of SM in a post-HSCT patient cohort composed exclusively of adult patients with SAA.

## Materials and methods

### Patients and transplantation procedure

**Fig. 1:**
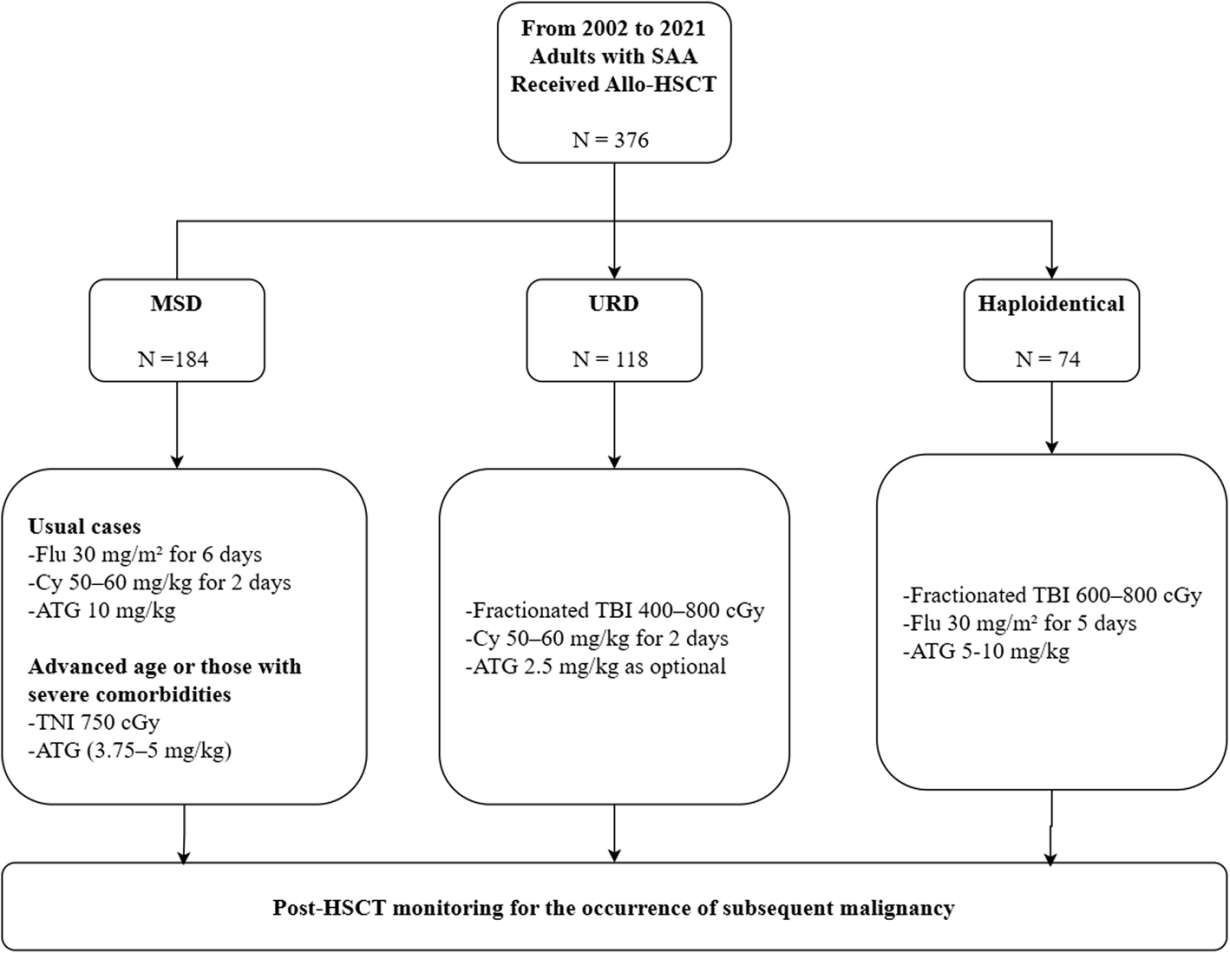
Patient configuration and allo-HSCT procedure. Allo-HSCT = Allogeneic hematopoietic stem cell transplantation; ATG = Anti-thymocyte globulin; Cy = Cyclophosphamide; Flu = Fludarabine; MSD = Matched sibling donor; SAA = Severe aplastic anemia; TBI = Total body irradiation; TNI = Total nodal irradiation; URD = Unrelated donor

This was a single-center retrospective cohort study conducted on adult patients with SAA who underwent allo-HSCT between 2002 and 2021. A total of 376 patients were included in the study. Patients were eligible for first-line allo-HSCT if they were 50 years of age or younger, had a good physical and functional status, and had a human leukocyte antigen (HLA)-matched (8 out of 8) sibling donor (MSD). Allo-HSCT was performed if patients failed one or more courses of IST or were deemed to be in urgent need of transplantation. In the absence of an MSD, an HLA-matched unrelated donor (URD) (≥ 6 out of 8 allele-matched) or a haploidentical donor was used. In MSD and URD HSCT, the bone marrow (BM) is the preferred stem cell source, although peripheral blood (PB) stem cells are sometimes used based on donor preference. The allo-HSCT regimen is shown in Fig. 1. Further details of this procedure are provided in previous publications [13, 14]. This study was approved by the Institutional Review Board of the Catholic University of Korea (Seoul, Republic of Korea). Approval number: KC24RASI0690. All procedures involving patient participation were conducted in accordance with the Declaration of Helsinki.

### Definitions

AA diagnosis and disease severity classification were based on criteria established by Camitta et al. [15]. Acute and chronic graft-versus-host disease (GVHD) were diagnosed and classified according to previously published diagnostic criteria [16, 17]. Primary and delayed graft failure were defined as failure of neutrophil engraftment on day 28 and irreversible absolute neutrophil count < 0.5 × 10^9^/L or platelet count < 20 × 10^9^/L in previous donor engraftment, respectively. Other definitions of transplant-related outcomes were consistent with previously published reports [13, 14]. The SM types were classified according to the Surveillance, Epidemiology, and End Results (SEER) database [18]. SM is divided into two categories: (1) hematologic malignancies, such as post-transplant lymphoproliferative disorder (PTLD), myelodysplastic neoplasm (MDS), and acute myeloid leukemia (AML), and (2) solid tumors, which include cancers of the skin, thyroid, liver, and gastrointestinal tract. SEER classifies tumors according to their site of origin and extent of spread. Tumors are classified as ‘localized’ if they are confined to the original site, ‘regional’ if they spread to nearby lymph nodes or tissues, or” distant’ if they spread to other organs. The same criteria were used to assess the initial extent of SM in all study patients. Carcinoma in situ and basal cell carcinoma of the skin were not considered SM in this study.

### Study objectives

The primary objective of this study was to estimate the cumulative incidence and determine the risk factors for SM in adult patients with SAA who underwent allo-HSCT. Additionally, the impact of SM on post-transplant survival was investigated. A comparative analysis with the general Korean population was performed to gain a more comprehensive understanding of the incidence of SM. Using data from the Korea Central Cancer Registry [19], we calculated the observed-to-expected (O/E) ratio (standardized incidence ratio) and the excess absolute risk (EAR) of malignancies in post-HSCT patients with SAA. As the most recent Korean population data are from 2021, comparisons with the general population were limited to SM cases that occurred up to 2021.

### Statistical analysis

Baseline and transplant-related categorical variables were described as frequency counts with percentages, whereas continuous variables were summarized as medians with interquartile ranges (IQR). Survival outcomes were estimated using Kaplan–Meier estimates. The occurrence of SM and other transplantation complications, such as graft failure, acute/chronic GVHD, cytomegalovirus (CMV) viremia, CMV disease, and hemorrhagic cystitis, was estimated using cumulative incidence estimates with 95% confidence intervals (CI). Competing risks for post-HSCT events included death from any cause, and graft failure for GVHD and primary graft failure for delayed graft failure were considered additional competing risks. The Fine-Gray sub-distribution hazard model was used to analyze the risk factors for SM occurrence. Time-dependent factors such as GVHD occurrence were treated as time-dependent variables. Survival was compared according to SM using the Mantel-Byar test [20], which is appropriate for comparing survival in cases with time-dependent covariates. A Simon-Makuch plot was used to visually represent survival differences over time [21]. A time-dependent Cox model was used to estimate the risk of post-HSCT survival due to the occurrence of SM. All time-dependent variables were measured on the day of stem cell infusion. In comparison with the general population, the expected number of cancers was calculated using population data by applying age-, sex-, and calendar year-specific incidence rates to the appropriate person-years at risk. Person-years at risk were calculated from the date of transplantation until the date of the last follow-up, death, or SM diagnosis, whichever occurred first. A schematic representation of the expected incidence of malignancy is shown in Supplementary Table S1. The EAR was calculated by subtracting the expected number of cancers from the observed number and dividing it by person-years at risk. All statistical analyses were performed with R version 4.2.2 (R Foundation for Statistical Computing, Vienna, Austria), and a *p*-value of < 0.05 was considered statistically significant.

## Results

### Baseline and transplant-related characteristics

The baseline and transplant-related characteristics are shown in Table 1. The median age of the patients was 34 years (interquartile range [IQR]: 27–45 years), and 37.5% of the patients were older than 40 years at the time of allo-HSCT. A total of 109 (34.3%) patients were diagnosed with very severe aplastic anemia (VSAA). One hundred and ninety-five (51.9%) patients received at least one course of IST before allo-HSCT. Non-radiation- and radiation-based conditioning regimens were used in 41.8% and 58.2% of the patients, respectively. For in vivo T-cell depletion, 43.9% of patients received 2.5–5 mg/kg anti-thymocyte globulin (ATG) and 41.0% of patients received 10 mg/kg ATG. MSD, URD, and haploidentical HSCT were performed in 48.9%, 31.4%, and 19.7% of the patients, respectively. The proportion of fully HLA-matched donors (eight out of eight allele-matched donors) was 71.0%. The graft sources were 41.8% BM, 54.3% PB, and 4.0% BM plus PB. None of the patients in our cohort had a history of malignancy prior to HSCT. After allo-HSCT, the cumulative incidence of primary and delayed graft failure was 0.9% (95% CI: 0.2%-2.3%) on day 28 and 7.5% (95% CI: 5.2%-10.9%) at 5 years, respectively. The cumulative incidence of grade II-IV acute and moderate to severe chronic GVHD was 26.2% (95% CI: 21.8–30.7) at day 100 and 15.0% (95% CI: 11.5–19.0) at 5 years, respectively. Overall survival (OS) and GVHD-free failure-free survival rates at 5 years were 91.5% (95% CI 88.2%-94.0%) and 71.3% (95% CI 66.4%-75.7%), respectively. The transplant-related outcomes according to donor type are described in Supplementary Table S2.

**Table 1:** Baseline and transplant-related characteristics of patients

Characteristics	Total *N* = 376
**Age, years (median, IQR)**	34 (27–45)
> 40 years (*N*, %)	141 (37.5)
**Male (*N*, %)**	170 (45.2)
**VSAA (*N*. %)**	129 (34.3)
**PNH clone positive (*N*, %)**	329 (87.5)
**IST history preceding HSCT (*N*, %)**	195 (51.9)
** ≥ 20U Transfusion before HSCT (*N*, %)**	316 (84.0)
** > 6 months from diagnosis to HSCT (*N*, %)**	272 (72.3)
**HCT-CI (median, IQR)**	2 (0–3)
≥ 3 (*N*, %)	132 (35.1)
**Female to male sex mismatch (*N*, %)**	145 (38.6)
**ABO mismatched (*N*, %)**	
Minor mismatch	69 (18.4)
Major mismatch	119 (31.6)
**HLA (8/8) mismatched (*N*, %)**	267 (71.0)
**Donor type (*N*, %)**	
Matched sibling	184 (48.9)
Unrelated	118 (31.4)
Haploidentical	74 (19.7)
**Stem cell source (*N*, %)**	
BM	157 (41.8)
PBSC	204 (54.3)
BM + PBSC	15 (4.0)
**Conditioning regimen (*N*, %)**	
Non-radiation-based	157 (41.8)
Radiation-based	219 (58.2)
TNI 750 cGy	23 (6.1)
Fractionated TBI 400-600 cGy	97 (25.8)
Fractionated TBI 800 cGy	99 (26.3)
**ATG dose (*N*, %)**	
Not used	57 (15.2)
2.5-5 mg/kg	165 (43.9)
10 mg/kg	154 (41.0)
**Transplantation outcomes (%, 95% CI)**^**a**^	
Graft failure	
Primary	0.9 (0.2–2.3)
Delayed	7.5 (5.2–10.9)
Acute grade II-IV GVHD	26.2 (21.8–30.7)
≥ Moderate chronic GVHD	15.0 (11.5–19.0)
CMV DNAemia	40.6 (95.6–45.5)
CMV disease	7.2 (4.9–10.1)
H.cystitis	7.7 (5.3–10.7)
OS rates	91.5 (88.2–94.0)
GFFS rates	71.3 (66.4–75.7)

*ATG* Anti-thymocyte globulin, *BM* Bone marrow, *CI* Confidence interval, *CMV* Cytomegalovirus, *GFFS* Graft-versus-host disease-free, failure-free survival, *GVHD* Graft-versus-host disease, *HCT-CI* Hematopoietic Cell Transplantation-specific Comorbidity Index, *H. cystitis* Hemorrhagic cystitis, *HSCT* Hematopoietic stem cell transplantation, *HLA* Human leukocyte antigen, *IST* Immunosuppressive therapy, *OS* Overall survival, *PBSC* Peripheral blood stem cell, *PNH* Paroxysmal nocturnal hemoglobinuria, *SAA* Severe aplastic anemia, *TBI* Total body irradiation, *TNI* Total nodal irradiation, *VSAA* Very severe aplastic anemia

^a^The incidence of primary graft failure and acute GVHD was estimated at 28 and 100 days, respectively. The incidence of other outcomes was estimated at five years

### Incidence and type of SMs

**Fig. 2:**
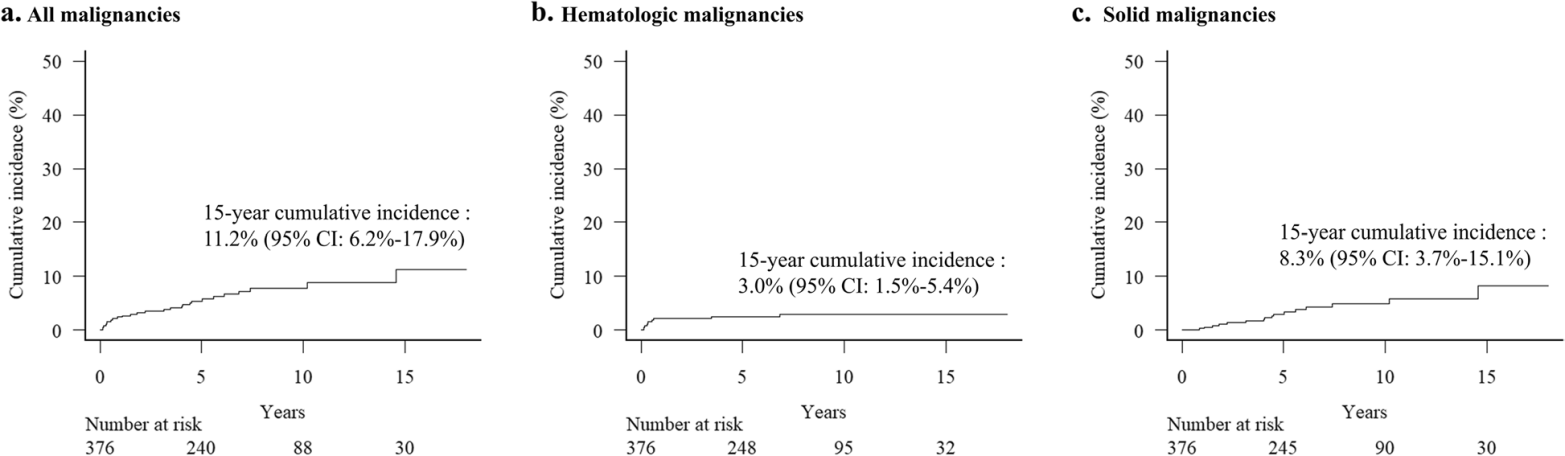
Cumulative incidence of (**a**) all types of subsequent malignancies, (**b**) hematologic malignancies, and (**c**) solid malignancies after hematopoietic stem cell transplantation. CI = confidence interval

The median follow-up duration of survivors was 6.7 years. During the observation period, 31 cases (8.2%) of SM were observed; one patient was diagnosed with three primary malignancies and another patient with two primary malignancies. The types and stages of SMs are listed in Table 2. Among the SMs, 32.3% had hematologic malignancies and 67.7% had solid malignancies. The frequencies of hematological malignancies were as follows: PTLD (16.1%), MDS (6.5%), and one case each of diffuse large B-cell lymphoma, NK/T-cell lymphoma, and AML (3.2%). The most common solid malignancy was thyroid cancer (25.8%), followed by stomach cancer (12.9%), oral cavity and pharyngeal cancer (9.7%), esophageal cancer (6.5%), colon cancer, bladder cancer, skin cancer, and cervical cancer ( 3.2%). Among hematologic malignancies, patients with NK/T-cell lymphoma have limited stages confined to the skin. Among solid malignancies, distant metastases were found in cases of thyroid, esophageal, bladder, and cervical cancers. Patients with stomach cancer are diagnosed at limited stages. Considering post-transplant death as a competing risk, the 15-year cumulative incidence of all SMs was 11.2% (95% CI: 6.2%-17.9%), and the estimates for hematologic and solid malignancies were 3.0% (1.5%-5.4%) and 8.3% (3.7%-15.1%), respectively (Fig. 2). The incidence of hematological malignancies plateaued, with the majority occurring within the first year after allo-HSCT, whereas the incidence of solid malignancies steadily increased (Fig. 2 and Supplementary Table S3).

**Table 2:** Incidence, types, and stage of subsequent malignancies

		**SEER summary stage**		
**Type (Total *N* = 31)**	***N* (%)**	**Localized**	**Regional**	**Distant**
Hematologic malignancy (*N* = 10)				
PTLD	5 (16.1)			5
MDS	2 (6.5)			2
DLBCL	1 (3.2)			1
NK/T cell lymphoma	1 (3.2)	1		
AML	1 (3.2)			1
Solid malignancy (*N* = 21)				
Thyroid	8 (25.8)	2	5	1
Stomach	4 (12.9)	4		
Oral cavity & Pharynx	3 (9.7)	1	2	
Esophagus	2 (6.5)			2
Colon	1 (3.2)	1		
Bladder	1 (3.2)			1
Cervical	1 (3.2)			1
Skin^a^	1 (3.2)	1		

*AML* Acute myeloid leukemia, *DLBCL* Diffuse large B-cell lymphoma, *MDS* Myelodysplastic neoplasm, *PTLD* Post-transplant lymphoproliferative disorder, *SEER* Surveillance, Epidemiology, and End Results

^a^Squamous cell carcinoma

### Risk factors for the incidence of SMs

The baseline and transplant-related variables had an impact on the occurrence of SM, as determined by the fine-gray sub-distribution hazard model (Table 3). In univariate analysis, the use of high-dose total body irradiation (TBI) during conditioning was identified as a significant variable associated with the occurrence of SM (*p* = 0.026). Patients with TBI doses > 600 cGy had a 15-year cumulative SM incidence of 17.6% (95% CI 8.9%-33.0%). Another significant variable in the univariate analysis was the Hematopoietic Cell Transplantation-specific Comorbidity Index (HCT-CI) score. In multivariate analysis, including age at HSCT and sex as adjusting covariates, not only HCT-CI and TBI dose but also ATG dose were identified as statistically significant predictors for the occurrence of SM. In this model, patients with a TBI dose > 600 cGy had a 4.60-fold (95% CI 1.37–15.46, *p* = 0.013) higher risk of developing SM than those with a lower TBI dose. Patients who received ATG dose > 5 mg/kg had a 3.45-fold (95% CI 1.04–11.53, *p* = 0.044) higher risk of SM than those with ≤ 5 mg/kg ATG dose. In addition, a time-dependent model was used to determine whether post-HSCT complications influenced the occurrence of SM (Supplementary Table S4). After adjustment for recipient age, sex, and HCT-CI, the occurrence of moderate to severe chronic GVHD was significantly associated with the development of SM (HR 2.41 [95% CI 1.02–5.67], *p* = 0.045). There was borderline significance in the incidence of acute GVHD grades II-IV (*p* = 0.084) and CMV DNAemia (*p* = 0.054).

**Table 3:** Univariate and multivariate Fine-Gray models for risk factors affecting the incidence of SMs following allogeneic HSCT

		**Univariate**		**Multivariate**	
**Variables**	**15-year** **Incidence**	**HR** **(95% CI)**	***p***^***a***^	**HR** **(95% CI)**	***p***^***a***^
Recipient age			0.664		0.754
< 40 years	12.8%	1		1	
≥ 40 years	8.1%	0.84 (0.38–1.86)		1.15 (0.48–2.73)	
Recipient sex			0.584		0.551
Male	9.80%	1		1	
Female	11.70%	1.24 (0.57–2.71)		1.27 (0.58–2.82)	
Disease severity			0.607		
SAA	13.60%	1			
VSAA	5.80%	0.81 (0.37–1.80)			
IST history before HSCT			0.480		
No	6.70%	1			
Yes	14.70%	1.32 (0.61–2.82)			
HCT-CI			0.017		0.030
0–2	7.0%	1		1	
≥ 3	18.1%	2.58 (1.18–5.63)		2.35 (1.09–5.08)	
Donor type			0.359		
Matched sibling	7.80%	1			
Unrelated	15.50%	1.76 (0.80–3.85)			
Haploidentical	6.90%	1.20 (0.38–3.79)			
Fludarabine used			0.414		
No	15.50%	1			
Yes	8.10%	0.74 (0.35–1.53)			
TBI dose			0.026		0.013
≤ 600 cGy	7.00%	1		1	
> 600 cGy	17.60%	2.31 (1.11–4.83)		4.60 (1.37–15.46)	
ATG dose			0.267		0.044
≤ 5 mg/kg	11.80%	1		1	
> 5 mg/kg	11.10%	1.51 (0.73–3.15)		3.45 (1.04–11.53)	

*ATG* Antithymocyte globulin, *CI* Confidence interval, *HCT-CI* Hematopoietic cell transplantation-specific comorbidity index, *HR* Hazard ratio, *HSCT* Hematopoietic stem cell transplantation, *IST* Immunosuppressive therapy, *SAA* Severe aplastic anemia, *SM* Subsequent malignancies, *TBI* Total body irradiation, *VSAA* Very severe aplastic anemia

^a^P values were calculated using the likelihood-ratio test

### Survival after SMs

**Fig. 3:**
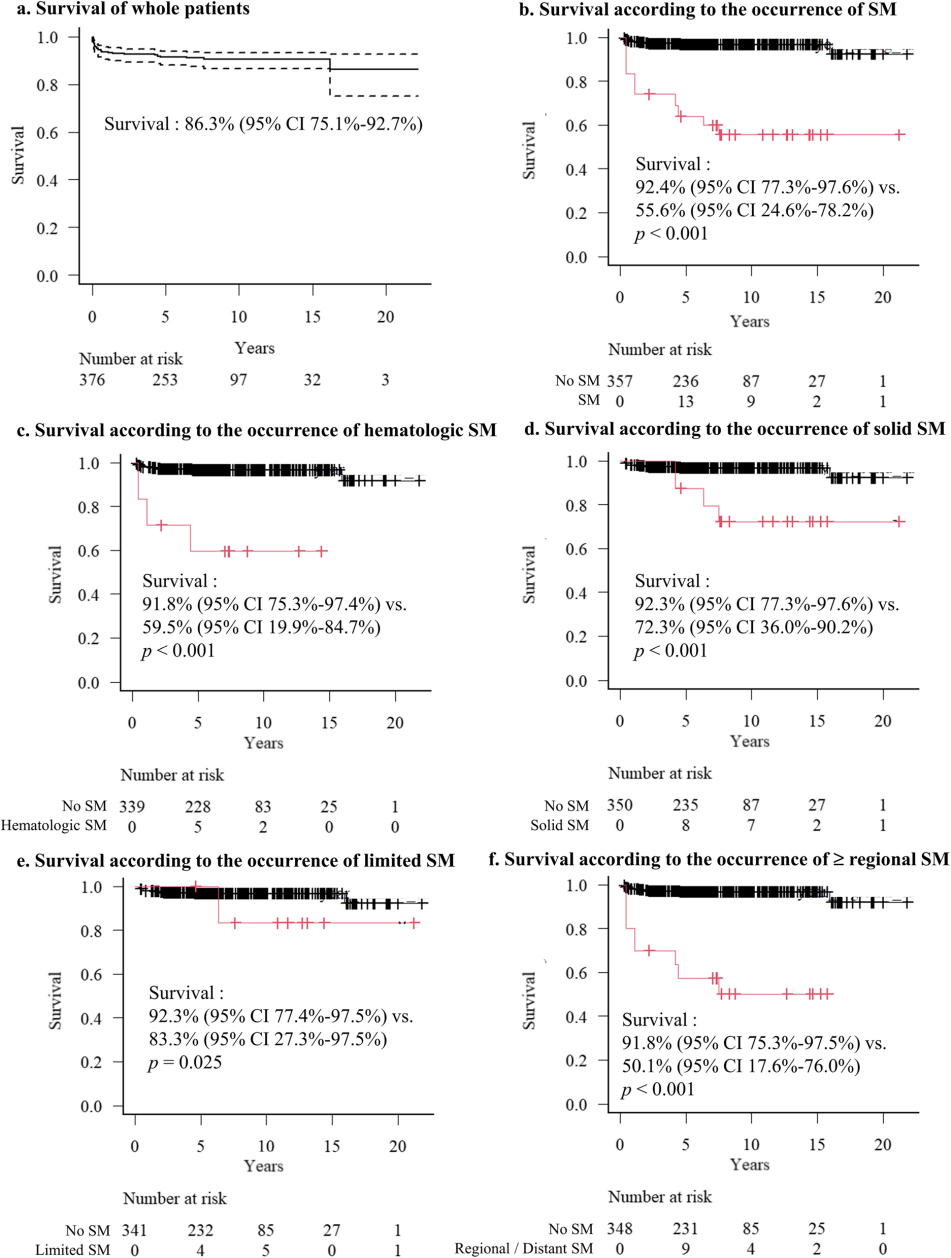
**a** Kaplan–Meier curve for overall survival in all patients. The other panels are Simon-Makuch plots for overall survival according to the occurrence of **b** whole SM, **c** hematologic SM, **d** solid SM, **e** limited SM, **f** ≥ regional SM. Red lines mean the SM development groups. *P* values are calculated using the Mantel-Byar test. CI = confidence interval; SM = subsequent malignancy

Post-transplant OS in this cohort was 86.3% (95% CI 75.1%-92.7%) of survival rate after HSCT (Fig. 3a). Figures 3B-3F visualized the OS according to the occurrence of SM using the Simon-Makuch method (setting the landmark date as 3 months). The presence of SM significantly affected OS after HSCT (Fig. 3b). In a time-dependent Cox regression model, the hazard ratio for OS according to the development of SM was 16.25 (95% CI 5.96–44.32). Patients with hematologic and solid malignancies demonstrated significantly worse OS compared with non-SM patients. Specifically, hematological malignancies were associated with worse long-term OS (59.5%, 95% CI 19.9%-84.7%) than solid malignancies (72.3%, 95% CI 36.0%-90.2%) (Fig. 3c and 3d). Regarding the summary stage, patients diagnosed with limited-stage SM had a relatively good prognosis, although a statistically significant difference remained compared to non-SM patients (Fig. 3e). Only one patient with limited-stage SM died; this patient had lung GVHD with subsequent respiratory failure. For patients with regional spread or metastatic SM, long-term OS was poor with 50.1% (95% CI 17.6%-76.0%) (Fig. 3f).

### Comparison with the general population

Table 4 summarizes the incidence of malignancy after allo-HSCT in patients with SAA compared to the age-, sex-, and calendar-year-matched general population using data derived from the Korea Central Cancer Registry [19]. The highest O/E ratio was 8.40 in the first year following transplantation, followed by 3.28, 2.69, 2.34, and 3.54 at one–five years, five-ten years, ten years, and beyond, respectively. Overall, a 3.54-fold increase in cancer incidence was identified in comparison with the age-, sex-, and year-matched general population. The absolute increase in the cancer incidence per 100 person-years was 0.85 cases. Owing to the small number of observed cases, it was not possible to approximate the O/E ratio estimates with a statistical distribution; thus, a CI for the O/E ratio was not calculated.

**Table 4:** Observed and expected cases of subsequent malignancies, calculated from population data, from the time of HSCT

	**Years from HSCT**				
	**0–1**	**1–5**	**5–10**	**10-**	**Total**
Person-years at risk	376	1131	697	260	2464
Observed	8	11	7	3	29
per 100 person-years	2.13	0.97	1.00	1.15	1.18
Expected	0.95	3.35	2.60	1.28	8.18
per 100 person-years	0.25	0.30	0.37	0.49	0.33
O/E	8.40	3.28	2.69	2.34	3.54
EAR, per 100 person-years	1.87	0.68	0.63	0.66	0.85

*EAR* Excess absolute risk, *HSCT* Hematopoietic stem cell transplantation, *O/E* Observed-to-expected ratio

## Discussion

Advances in transplantation techniques, donor selection, and supportive care have significantly improved post-HSCT survival in patients with SAA and have expanded the feasibility of HSCT. However, these advances have also drawn greater attention to post-transplant complications in SAA, particularly the occurrence of SM, for which data remain limited. In the current study, SM occurred in approximately 11% of patients who survived long-term post-transplant follow-up (Fig. 2). Given that our cohort had a median age of 34 years, with 37.5% of patients aged > 40 years (Table 1), this finding is crucial and should not be overlooked. SM has been observed in various organs, including both hematological and solid manifestations, and it has a profound detrimental impact on post-HSCT survival, especially in invasive cases. Moreover, the current study showed that the incidence of SM in the posttransplant SAA patient cohort was significantly higher than that in the general population of the same region.

In this study, approximately one-third of the SM cases were hematologic malignancies. The incidence of these hematologic malignancies increased sharply within the first year after HSCT and then reached a plateau (Fig. 2 and Table 5), which aligns with a previous report [7]. This early occurrence pattern may be attributed to the engraftment process and the use of immunosuppressive drugs, which impair T-cell function and create conditions that favor the proliferation of abnormal hematological cells until immunological regulation is re-established [22]. The most common hematological SM in this study was PTLD, a common finding in most studies. However, the incidence in our cohort (5 of 376, 1.3%) was lower than that in other studies, which have reported rates up to 10% in reduced-intensity conditioning (RIC) settings [10, 22–24]. Previous studies have shown that the occurrence of PTLD increases in patients who receive the RIC regimen or a regimen containing a high dose of ATG [24–26]. In this study, most transplants were performed using a non-myeloablative, reduced-toxicity regimen, which is a standard treatment strategy. Additionally, transplants using only total nodal irradiation (TNI) 750 cGy and ATG were performed, as this regimen was previously shown to be a viable approach [13]. This discrepancy may be due to the benign nature of SAA. Also, the increased incidence of PTLD in the RIC regimen may be explained by the fact that patients who receive the RIC regimen tend to be older and have higher baseline Epstein-Barr virus (EBV) viral loads [24], rather than an association of PTLD with the regimen itself. Since most patients in this study were mostly younger (< 40 years old), this may explain the lower incidence of PTLD. In addition, reports suggest a higher incidence of PTLD when alternative stem cell sources, such as unrelated donors or cord blood, are used [11, 27]. HSCT is typically considered in SAA when a sibling donor is available; in our study, almost half of the patients (49%) underwent MSD HSCT.

In contrast to hematologic malignancies, the incidence of solid SMs does not plateau but instead shows a continuous upward trend. This pattern is likely driven by a natural increase in solid malignancy rates with age [7]. Thyroid and gastric cancers are the most common solid SMs. While post-HSCT pathological mechanisms may contribute to their occurrence, it is plausible that the high incidence rates are due to the widespread screening using ultrasound and endoscopy in this region, making these cancers easier to identify [28, 29]. Reflecting on these active cancer surveillance efforts, all 4 cases of gastric cancer in this study were detected at a limited stage. Cancers such as skin, oral cavity, and colon cancers, which occur in areas commonly affected by GVHD, support the evidence for a correlation between GVHD and SM, as highlighted by Danylesko et al. [30]. They reported a significant association between chronic GVHD and the development of SM, particularly in squamous cell carcinoma. In the current study, chronic GVHD was identified as a significant time-dependent variable associated with the occurrence of SM. However, due to the small number of patients with squamous cell carcinoma (three patients), we were unable to statistically confirm the correlation between chronic GVHD and the specific histological type of solid malignancy.

In addition to chronic GVHD, this study identified significant associations between SM occurrence and factors such as high HCT-CI (HR, 2.4), high-dose TBI (HR, 4.6), and high-dose ATG (HR, 3.5). These findings align with those of previous studies on the impact of high-dose TBI and ATG on SM risk [9, 11]. Furthermore, the predictive power of the comorbidity index for posttransplant SM is intriguing. Acute GVHD (*p* = 0.074 and *p* = 0.084 in univariate and multivariate analyses, respectively) and CMV DNAemia (*p* = 0.040 and *p* = 0.054 in univariate and multivariate analyses, respectively) also showed borderline significance for SM development, although their independent effects remain unclear because of collinearity with other transplant variables. However, further studies are required to confirm these findings.

The current study demonstrated that SM occurrence had a significant negative impact on post-HSCT survival with an HR of 16.3. As expected, patients diagnosed at a limited stage had favorable outcomes, with only one death unrelated to SM, whereas those with regional or distant stages had notably poor prognoses (Fig. 3). Although hematologic malignancies predominated in the early post-transplantation period, the elevated cancer incidence persisted when compared to the matched general population (Table 4). These findings emphasize the need for comprehensive monitoring. For example, Bhatia et al. recommended an annual history and physical examination of the oral cavity, uterine cervix, external genitalia, and full skin, as well as an annual breast examination, mammogram, breast MRI (beginning 8 years after radiation or age 25 years, whichever occurs first), colonoscopy every 5 years (beginning 10 years after radiation or age35), and thyroid ultrasound [4]. A more thorough protocol, including gastroscopy, is required. In addition, lifestyle management and cancer prevention protocols to reduce cancer risk (such as avoidance of tobacco and alcohol consumption, use of sunscreen, and HPV vaccination) should be actively implemented in post-HSCT SAA survivors [6].

The novelty of this study is that it follows the development of SM in a large number of patients with SAA. To the best of our knowledge, no other study has exclusively tracked the development of SM after HSCT in patients with SAA in Asia, and no study focusing on patients with SAA has been conducted worldwide. Nevertheless, the small number of patients (*n* = 376) may have limited the ability to determine the exact incidence of SM and its risk factors. Another limitation of this study is that the follow-up period may not have been sufficiently long, as the development of SM may take a long time. However, it should be noted that patients included in this study were followed up at least every 6 months if they did not die and had regular medical histories, physical examinations, and national cancer screening programs [29]. Other limitations of the study include the lack of EBV serology data and the low number of SM cases, which prevented us from identifying individual risk factors for each cancer type. Future studies should be conducted on a larger scale with longer follow-up periods. Additionally, because cancer incidence varies by region and race, further research in diverse populations is required. Incorporating genetic information to identify links to SM development could also be a valuable topic for future research.

In conclusion, the current study demonstrated that SM development in patients with SAA undergoing allo-HSCT is a significant complication influenced by factors such as the dose of ATG or TBI in the conditioning regimen and the occurrence of moderate-to-severe chronic GVHD. SM, particularly in advanced stages, has a marked adverse effect on post-HSCT survival. Additionally, post-HSCT patients with SAA exhibit a higher incidence of long-term cancer than the matched general population. These findings should guide conditioning regimen planning, particularly for high-risk cancer patients, and underscore the need for thorough monitoring and management protocols to track post-transplant SM development.

## Supplementary Information

Supplementary Material 1.

## Data Availability

No datasets were generated or analysed during the current study.
