# Supplementary material for: Incidence and outcomes of subsequent malignancy after allogeneic hematopoietic stem cell transplantation in adult patients with severe aplastic anemia
Source: Blood Res. 2024 Dec 24;59(1):44. doi: 10.1007/s44313-024-00046-2 (PMC11668717; doi:10.1007/s44313-024-00046-2)
Supplement: Supplementary file 1 — Supplementary Material 1. [file 44313_2024_46_MOESM1_ESM.docx]

**Supplementary Table S1. Calculation of the expected incidence of malignancy**

|  |  | Year |  |  |  |  |  |  |  |  |  |
| --- | --- | --- | --- | --- | --- | --- | --- | --- | --- | --- | --- |
|  | Case, Sex | 2004 | 2005 | 2006 | 2007 | 2008 | 2009 | 2010 | 2011 | 2012 | 2013 |
| Person-Year | 1, male | 0.5 | 1 | 1 | 1 | 0.6 |  |  |  |  |  |
| Age group |  | [30,35) | [30,35) | [30,35) | [30,35) | [30,35) |  |  |  |  |  |
| Incidence |  | 0 | 0 | 0 | 0 | 1 |  |  |  |  |  |
| Expected |  | 22.1 | 47.5 | 49.7 | 54.4 | 35.28 |  |  |  |  |  |
| Person-Year | 2, female |  | 0.4 | 1 | 1 | 1 | 1 | 1 | 1 | 1 | 0.4 |
| Age group |  |  | [20,25) | [20,25) | [20,25) | [25,30) | [25,30) | [25,30) | [25,30) | [25,30) | [30,35) |
| Incidence |  |  | 0 | 0 | 0 | 0 | 0 | 0 | 0 | 0 | 0 |
| Expected |  |  | 12.72 | 32.8 | 35.2 | 93 | 102.9 | 113.2 | 115.8 | 124.1 | 92.32 |
| Person-Year | 3, male | 0.1 | 1 | 1 | 1 | 1 | 1 | 1 | 1 | 1 | 0.2 |
| Age group |  | [30,35) | [30,35) | [35,40) | [35,40) | [35,40) | [35,40) | [35,40) | [40,45) | [40,45) | [40,45) |
| Incidence |  | 0 | 0 | 0 | 0 | 0 | 0 | 0 | 0 | 0 | 1 |
| Expected |  | 4.42 | 47.5 | 84.6 | 90.1 | 97 | 109.1 | 114 | 197.2 | 196.6 | 40.1 |
| Person-Year | 4, female |  |  | 0.2 | 1 | 1 | 1 | 1 | 0.3 |  |  |
| Age group |  |  |  | [45,50) | [50,55) | [50,55) | [50,55) | [50,55) | [50,55) |  |  |
| Incidence |  |  |  | 0 | 0 | 0 | 0 | 0 | 1 |  |  |
| Expected |  |  |  | 88.5 | 550.4 | 599.1 | 645 | 699.4 | 217.35 |  |  |
| Total Person-years | | 0.6 | 2.4 | 3.2 | 4 | 3.6 | 3 | 3 | 2.3 | 2 | 2 |
| Total Incidence | | 0 | 0 | 0 | 0 | 1 | 0 | 0 | 1 | 0 | 1 |
| Total Expected | | 26.52 | 107.72 | 255.6 | 730.1 | 824.38 | 857 | 926.6 | 530.35 | 320.7 | 132.42 |

Table 1 presents hypothetical cases 1-4. Cases 1, 3, and 4 involve patients who developed subsequent malignancies, while case 2 represents a deceased patient. For each patient, we calculated the person-years during the follow-up period for the relevant calendar year, identified the appropriate age group for each year, and extracted the expected cancer incidence by year, sex, and age group from the Korean Cancer Registry. The expected incidence rate in the table is expressed as the number of cases per 100,000 person-years. Using this method, we compared the actual incidence with the expected incidence across all patients

**Supplementary Table S2. Transplant-related outcomes by donor types**

| **Characteristics** | **Overall** | **MSD** | **URD** | **Haplo** | ***p*^b^** |
| --- | --- | --- | --- | --- | --- |
|  | **Cumulative incidence or rate (%, 95% CI)^a^** | | | |  |
| Number of patients | 376 | 184 | 118 | 74 |  |
| Graft failure |  |  |  |  |  |
| Primary | 0.9 (0.2-2.3) | 0 | 0 | 4.4 (0.1–11.4) | < 0.001 |
| Delayed | 7.5 (5.2-10.9) | 14.6 (9.8-20.4) | 0 | 1.5 (0.1–6.5) | < 0.001 |
| Acute grade II-IV GVHD | 26.2 (21.8-30.7) | 10.4 (6.5-15.3) | 45.8 (36.5-54.5) | 34.2 (23.5-45.1) | < 0.001 |
| ≥ Moderate chronic GVHD | 15.0 (11.5-19.0) | 7.2 (4.0-11.6) | 25.4 (17.4-34.3) | 20.2 (11.6-30.4) | < 0.001 |
| CMV DNAemia | 40.6 (95.6-45.5) | 38.9 (31.8-45.9) | 40.7 (31.7-49.4) | 44.6 (33.0-55.6) | 0.925 |
| CMV disease | 7.2 (4.9-10.1) | 3.8 (1.7-7.3) | 7.6 (3.7-13.3) | 14.9 (7.9-24.0) | 0.009 |
| H.cystitis | 7.7 (5.3-10.7) | 6.0 (3.2-10.1) | 8.5 (4.3-14.4) | 10.9 (5.0-19.2) | 0.422 |
| OS rates | 91.5 (88.2-94.0) | 94.4 (89.9-97.0) | 87.9 (80.3-92.6) | 90.5 (81.1-95.4) | 0.049 |
| GFFS rates | 71.3 (66.4-75.7) | 77.1 (70.2-82.6) | 59.1 (49.6-67.4) | 76.9 (65.4-84.9) | 0.001 |

a. The incidence of primary graft failure and acute GVHD was estimated at 28 days, and 100 days, respectively. The incidence of other outcomes was estimated at 5 years. b. P values of OS rates, GFFS rates are estimated by log-rank test. The others are estimated by Fine-Gray test. CI = confidence interval; CMV = cytomegalovirus; GFFS = graft-versus-host disease-free failure-free survival; GVHD = graft-versus-host disease; Haplo = haploidentical related donor; H.cystitis = hemorrhagic cystitis; MSD = matched sibling donor; OS = overall survival; URD = matched unrelated donor.

**Supplementary Table S3. Incidence of subsequent malignancy per year after transplantation until 16 years after HSCT**

| **Post-HSCT Year** | **0-1** | **1-2** | **2-3** | **3-4** | **5-6** | **6-7** | **7-8** | **8-9** | **9-10** | **10-11** | **11-12** | **12-13** | **13-14** | **14-15** | **15-16** |
| --- | --- | --- | --- | --- | --- | --- | --- | --- | --- | --- | --- | --- | --- | --- | --- |
| N. at risk | 376 | 349 | 340 | 316 | 292 | 253 | 207 | 165 | 140 | 115 | 97 | 87 | 71 | 55 | 45 |
| Death | 24 | 3 | 1 | 1 | 3 | 0 | 1 | 1 | 0 | 0 | 0 | 0 | 0 | 0 | 0 |
| SM | 8 | 4 | 1 | 2 | 4 | 0 | 4 | 1 | 0 | 1 | 2 | 0 | 0 | 0 | 1 |
| Hematologic | 8 | 0 | 0 | 1 | 0 | 0 | 1 | 0 | 0 | 0 | 0 | 0 | 0 | 0 | 0 |
| Solid | 0 | 4 | 1 | 1 | 4 | 2 | 3 | 1 | 0 | 1 | 2 | 0 | 0 | 0 | 1 |

**Supplementary Table S4. Time-dependent subdistribution hazard model for the occurrence of subsequent malignancy**

|  | **Univariate** | | **Age, sex, and HCT-CI adjusted model** | |
| --- | --- | --- | --- | --- |
| **Variables** | **HR (95% CI)** | ***P*^a^** | **HR (95% CI)** | ***p*^a^** |
| Acute grade II-IV GVHD | 1.99 (0.93-4.24) | 0.074 | 2.05 (0.91-4.63) | 0.084 |
| ≥ Moderate chronic GVHD | 2.36 (1.03-5.42) | 0.043 | 2.41 (1.02-5.67) | 0.045 |
| CMV DNAemia | 2.30 (1.04-5.09) | 0.040 | 2.20 (0.99-4.92) | 0.054 |
| CMV disease | 1.67 (0.62-4.54) | 0.312 | 1.70 (0.63-4.58) | 0.292 |
| H.cystitis | 1.11 (0.40-3.05) | 0.845 | 1.09 (0.39-3.06) | 0.873 |

a. P values are calculated by Wald test. CI = confidence interval; CMV = cytomegalovirus; GVHD = graft-versus-host disease; HCT-CI = Hematopoietic Cell Transplantation-specific Comorbidity Index; H.cystitis = hemorrhagic cystitis; HR = hazard ratio.
